# Microglia exacerbate white matter injury via complement C3/C3aR pathway after hypoperfusion

**DOI:** 10.7150/thno.35841

**Published:** 2020-01-01

**Authors:** Lin-Yuan Zhang, Jiaji Pan, Muyassar Mamtilahun, Yuan Zhu, Liping Wang, Ashwin Venkatesh, Rubing Shi, Xuanqiang Tu, Kunlin Jin, Yongting Wang, Zhijun Zhang, Guo-Yuan Yang

**Affiliations:** 1 Department of Neurology, Ruijin Hospital School of Medicine, and Med-X Research Institute and School of Biomedical Engineering, Shanghai Jiao Tong University, Shanghai, China; 2 Department of Physiology, Development and Neuroscience, University of Cambridge, Cambridge, United Kingdom; 3 Department of Pharmacology and Neuroscience, University of North Texas Health Science Center, Fort Worth, TX 76107, USA

**Keywords:** chronic cerebral hypoperfusion, complement, inflammation, microglia, white matter injury

## Abstract

Microglial activation participates in white matter injury after cerebral hypoperfusion. However, the underlying mechanism is unclear. Here, we explore whether activated microglia aggravate white matter injury via complement C3-C3aR pathway after chronic cerebral hypoperfusion.

**Methods**: Adult male Sprague-Dawley rats (n = 80) underwent bilateral common carotid artery occlusion for 7, 14, and 28 days. Cerebral vessel density and blood flow were examined by synchrotron radiation angiography and three-dimensional arterial spin labeling. Neurobehavioral assessments, CLARITY imaging, and immunohistochemistry were performed to evaluate activation of microglia and C3-C3aR pathway. Furthermore, C3aR knockout mice were used to establish the causal relationship of C3-C3aR signaling on microglia activation and white matter injury after hypoperfusion.

**Results**: Cerebral vessel density and blood flow were reduced after hypoperfusion (*p<*0.05). Spatial learning and memory deficits and white matter injury were shown (*p<*0.05). These impairments were correlated with aberrant microglia activation and an increase in the number of reactive microglia adhering to and phagocytosed myelin in the hypoperfusion group (*p<*0.05), which were accompanied by the up-regulation of complement C3 and its receptors C3aR (*p<*0.05). Genetic deletion of *C3ar1* significantly inhibited aberrant microglial activation and reversed white matter injury after hypoperfusion (*p<*0.05). Furthermore, the C3aR antagonist SB290157 decreased the number of microglia adhering to myelin (*p<*0.05), attenuated white matter injury and cognitive deficits in chronic hypoperfusion rats (*p<*0.05).

**Conclusions**: Our results demonstrated that aberrant activated microglia aggravate white matter injury via C3-C3aR pathway during chronic hypoperfusion. These findings indicate C3aR plays a critical role in mediating neuroinflammation and white matter injury through aberrant microglia activation, which provides a novel therapeutic target for the small vessel disease and vascular dementia.

## Introduction

Chronic cerebral hypoperfusion is closely related to cognitive impairment and vascular dementia 1, 2. Increasing evidence indicates that chronic cerebral hypoperfusion could precede and contribute to white matter injury, which is an important pathological feature of vascular dementia 3, 4. Clinical studies have shown that white matter injury in vascular dementia is often more severe than that typically seen in Alzheimer's disease (AD) and Lewy body dementia 5-7. In rodent, white matter damage is also a canonical feature of hypoperfusion 8, 9. To explore the underlying mechanism, the rat model of bilateral common carotid artery occlusion (BCCAO) and mouse model of bilateral common carotid artery stenosis (BCAS) were used to mimic white matter injury during vascular dementia in humans 9-11.

Previous studies have suggested that neuroinflammation plays a crucial role in white matter injury and cognitive dysfunction induced by chronic cerebral hypoperfusion. Microglia are resident immune cells of the CNS that maintain brain homeostasis 12. In pathological states, microglia could be activated to the detrimental phenotype which lead to tissue damage or transform into the protective phenotype secreting growth factors and promote tissue recovery. Their transcriptomic and proteomic profiles could be drastic different depending on the signals in the lesion microenvironment 13. Phenotypic changes in microglia correlate with the prognosis of autoimmune demyelination disorders 14, 15. Neuropathologic studies of white matter lesions have shown that robust neuroinflammatory responses are characterized by highly activated microglia 4, 16.

Accumulated evidence shows the complement system plays a critical role in microglia-mediated effects during physiological and pathological processes 17. The complement proteins C1q and C3 mediate synapse elimination during development and neurodegeneration through C3-dependent microglia phagocytosis 18-20. The classical complement pathway triggers microglia activation and induces early synaptic loss and dysfunction in AD 21. C3 deficiency suppresses microglia activation and protects against cognitive decline at later AD stages 22. The activation of the complement system is also involved in white matter injury in multiple sclerosis 23. C3a and C5a overexpression in the CNS aggravates the severity of cuprizone-induced white matter injury 24. Knocking out C5 alleviates white matter lesions in the corpus callosum after chronic cerebral ischemia 25. The complement cascade is also involved in acute cerebral vascular disease. Inhibiting the complement system reduces brain edema in the acute phase of hemorrhagic stroke and infarct volumes in acute ischemic stroke animals 26, 27. However, it is unclear whether and how complement-mediated microglia activation contributes to white matter injury after chronic cerebral hypoperfusion. We hypothesize that during chronic cerebral hypoperfusion, up-regulated complement C3 induces aberrant microglia activation and aggravates white matter injury through microglia movement to and phagocytosis of myelin. Our study could provide a novel therapeutic strategy for the small vessel disease and vascular dementia.

## Materials and Methods

**Figure 1:**
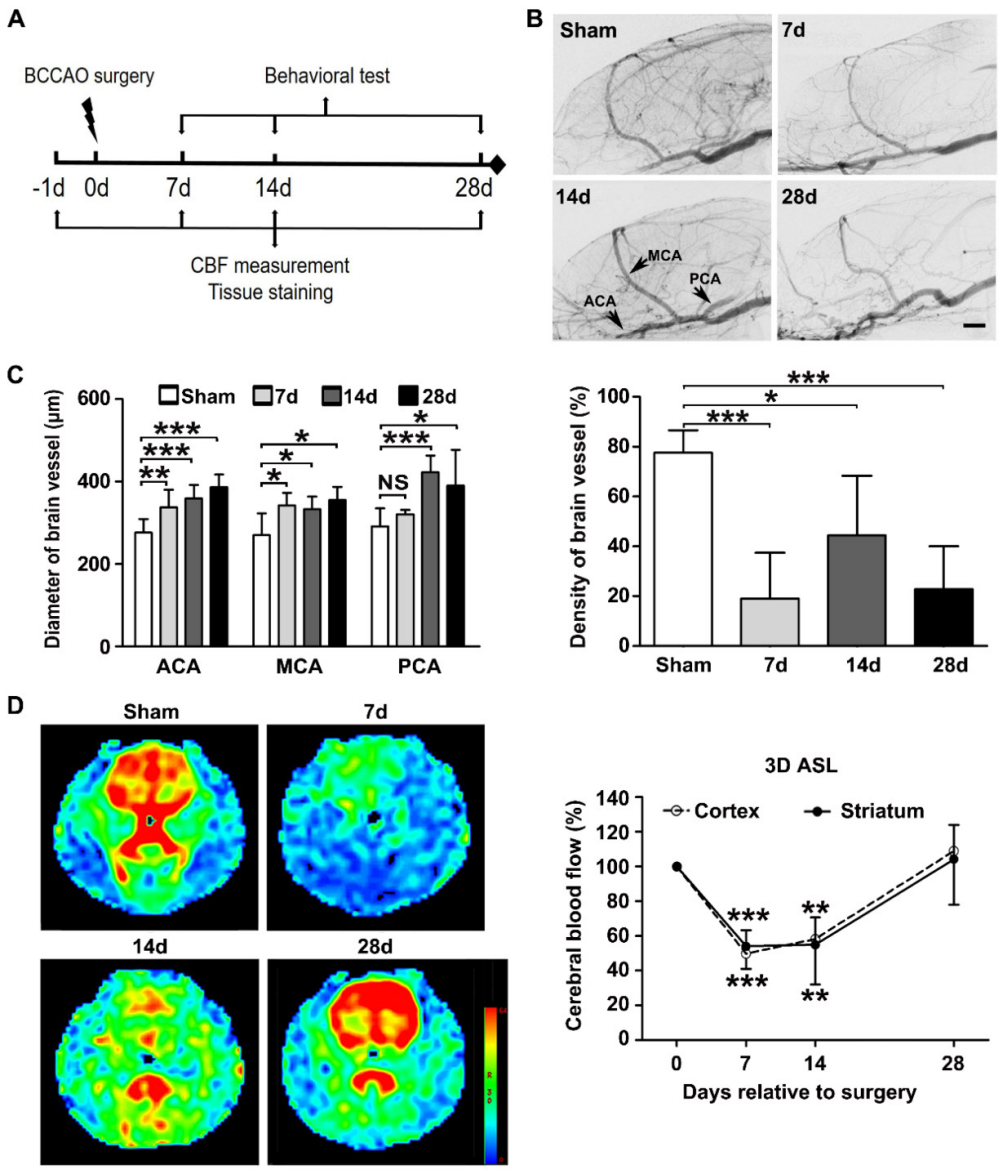
** Experimental design and BCCAO-induced cerebral vessel and dynamic cerebral blood flow changes. A** Experimental design. BCCAO surgery was performed at day 0, and the animals were sacrificed at day 7, 14, and 28 after BCCAO. **B** Representative images of SRA in control and BCCAO rats at day 7, 14, and 28 after surgery. n = 6-12 animals in each group. Scale bar=1 mm. **C** Quantification of arterial diameters and cerebral vascular density in control and BCCAO rats at day 7, 14, and 28 after surgery. **D** Representative 3D arterial spin labeling images in rats at baseline and day 7, 14, and 28 after BCCAO and dynamic cerebral blood flow changes in the cortex and striatum in BCCAO rats at day 7, 14, and 28 after surgery, relative to baseline levels. n = 7 animals. The data are shown as the mean ± SD. ***, *p*< 0.001; **, *p*< 0.01; *, *p*< 0.05; NS, not significant; the BCCAO group vs. the control group. 3D ASL: 3D arterial spin-labeled imaging; ICA: internal carotid artery; MCA: middle cerebral artery; PCA: posterior cerebral artery.

The animal experimental procedures were approved by the Institutional Animal Care and Use Committee (IACUC) of Shanghai Jiao Tong University, China. We performed all animal experiments in accordance with the ARRIVE guidelines. The experimental design is shown in **Figure 1A**. Cerebral vessel density and cerebral blood flow were determined by synchrotron radiation angiography (SRA) and three-dimensional arterial spin labeling at day 7, 14, and 28 after BCCAO. Neurobehavioral assessments were performed using the Morris water maze and novel object recognition tests. After C3aR antagonist treatment, immunostaining and behavioral studies were performed at day 14 and day 28. In C3aR knockout mice, immunohistochemical assessments were performed at day 28 after BCAS surgery.

### Animals

Adult male Sprague-Dawley rats weighing 250-300 g and C57BL/6 mice weighting 25 to 30 g (SLAC Laboratory Animals, Shanghai, China) were used in present study. Homozygous C3aR knockout mice (C.129S4-*C3ar1^tm1Cge^*/J, #005712, Jackson laboratory) weighting 25 to 30 g were used to evaluate C3-C3aR signaling on microglia activation and white matter injury in chronic cerebral hypoperfusion. Only male rodents were used in our study to exclude neuroprotection effect of estrogen on ischemic damage 28. Animals were housed in groups of 4 to 10, under a 12-hour light/12-hour dark cycle at 21 °C, 50% humidity. Food and water were available *ad libitum*. Animal group size for behavioral or immunological studies was determined according to our preliminary results. Power analysis was performed using a type I error rate of 0.05 and a power of 80% on a two-sided test. Animals were randomly assigned to sham-operated or chronic cerebral hypoperfusion surgery groups at the beginning of each experiment. Animals in the BCCAO or BCAS group were subjected randomly to drugs or vehicle treatment. Independent cohorts were used for synchrotron radiation angiography and three-dimensional arterial spin labeling. Animals were coded with numbers, and experimenters were blinded to the treatment assignment.

### Animal surgery

Rats subjected to permanent bilateral common carotid artery occlusion (BCCAO) surgery and mice subjected to bilateral common carotid artery stenosis (BCAS) surgery were conducted as described previously 10, 29. Briefly, the rats were anesthetized with 2% isoflurane in 0.5 L/min oxygen. Both common carotid arteries were ligated with two 5-0 sutures (Covidien, Mansfield, MA). Mice were anesthetized with 1.5% isoflurane in a 30% O2/68.5% NO mixture, 0.18mm diameter microcoils (Sawane Spring Co., Shizuoka, Japan) are placed at both common carotid arteries just below the carotid bifurcation. Sham animals underwent bilateral common carotid artery exposure but not artery occlusion or stenosis. SB290157 (Merck Millipore, Darmstadt, Germany, #559410), a specific C3a receptor antagonist 30, was diluted in sterile phosphate-buffered saline (PBS)/2% DMSO to a concentration of 1 nM. Intraperitoneal injections of SB290157 (1 mg/kg) or 2% DMSO (vehicle) were performed immediately after BCCAO and once a day for 28 days. Intraperitoneal injection of minocycline (MedChemExpress, Monmouth, NJ, #HY-17412), an inhibitor of microglial activation, at a dose of 25 mg/kg was performed immediately after BCCAO surgery and once a day for 28 days to attenuate microglia activation. Intraperitoneal injections of PLX3397 (MedChemExpress, Monmouth, NJ, #HY-16749), colony-stimulating factor 1 receptor (CSF1R) inhibitors at a dose of 1 mg/kg was performed immediately after BCAS surgery and twice a day for 28 days to eliminating microglia according to the instruction.

### Synchrotron radiation angiography

SRA was performed at beam line 13W at the Shanghai Synchrotron Radiation Facility (SSRF), Shanghai, China. The imaging parameters and procedures were described previously 31. Briefly, an angiographic tube (PE-10 catheter linked with PE-50 catheter) linked with a 10-mL syringe was carefully inserted from the distal external carotid artery to the bifurcation of the internal carotid artery and the external carotid artery for injecting contrast agent. The syringe was connected to a microinjection system consisting of a precise injection pump (Longerpump, Baoding, China). The energy of the X-ray was 33.2 KeV. Nonionic iodine contrast agent (300 mg/ml, Omnipaque, GE, Milwaukee, WI) was injected into the external carotid artery reversed at an injecting speed of 133.3 μl/s, with a resulting volume of 150 μl. The Charge Coupled Device (CCD) with 6.5 μm resolution (Photonic Science, East Sussex, UK) was placed 65 cm away from the animal. Ten consecutive images taken before contrast-enhancement were subtracted from raw images taken after injection to eliminate the background structure. The images obtained were then processed with MATLAB (Mathworks, Natick, MA) and stitched with Adobe Photoshop CS6 (Adobe, San Jose, CA).

### Magnetic resonance imaging

MRI experiments were conducted with a Discovery 750 3.0 T scanner with a small animal wrist coil (GE Healthcare, Milwaukee, WI). The animals were anesthetized with 7% chloral hydrate (0.3 ml/100 g) and placed in a supine position before scanning. The imaging parameters for the 3D ASL series were as follows: field of view = 120 mm × 120 mm, matrix = 512 (points) × 12 (arms); 15 slices (each 4 mm thick) were acquired in ascending order with no gap between slices; labelling duration = 1,650 ms; post-labeling delay = 1,025 ms; repetition time = 4,132 ms; echo time = 11 ms; number of excitations = 5; bandwidth = 62.5 kHz; scan duration = 14 min 14 s. Cerebral blood flow was automatically calculated using Functool 3D ASL (GE Medical Systems, Milwaukee, WI). For the quantification of cerebral blood flow, the following equation was used:


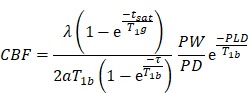


In the equation, T1b was T1 of blood (1,600 ms); T1g, T1 of gray matter (1,200 ms); α, the labelling efficiency (0.95); λ, the cortex-blood partition coefficient (0.9); t_sat_, the time of saturation performed before imaging (2,000 ms); τ, the labeling duration (1,500 ms); and PLD, the post labeling delay time (1,025 ms).

### Neurobehavioral assessments

Control and BCCAO rats treated with the C3aR antagonist or 2% DMSO underwent both the Morris water maze test and novel object recognition test on day 7, 14, 28. All tests were monitored and quantitatively analyzed by behavioral tracking system (Stoelting Co., Wood Dale, IL). The Morris water maze and novel object recognition tests were performed as previously described 32, 33. All trials were performed in a quiet room with indirect lighting. The Morris water maze test was performed in a circular tank with a diameter of 170 cm, containing water at approximately 20-22 ℃. The swimming pool was virtually divided into four equal quadrants. The edible pigment was used to opacity the water to camouflage the submerged platform. The hidden circular platform (9 cm in diameter) was submerged 1.5 cm below the water surface and remained fixed throughout the spatial acquisition trials.

The spatial acquisition trials were conducted over five consecutive days. Each trial had a maximum time of 2 min with an interval of 15 s between trials, four trials were completed per day. The animal was placed in one of four designated start positions in the maze, facing the sidewalls of the swimming pool. If the animal failed to reach the platform within 2 min, it was guided to the platform using a guide stick. After the animal reached the escape platform, it remained on the platform for 15 s before the beginning of the next trial. The escape latency was defined as the time that the animal spent to reach and climb onto the escape platform from the start position. On day 6, the probe test was conducted. The escape platform was removed from the pool and the animals could swim for 60 s. The percentage time spent in the target quadrant and the number of times that the animal crossed the zone where the platform formerly existed were recorded. Following probe test, Four-day working memory tests were performed. The platform was moved each day and each animal was given 2 trials per day (**Figure S4A**). Trial 1 was the sample trial to learn the new location of the platform, and trial 2 was performed for recall and the temporary memory measurement after a 15 seconds of inter-trial interval.

In the novel object recognition test, rats were placed in a 45 cm (long) × 45 cm (wide) × 45 cm (height) sized box, and were permitted to explore the testing area for 10 min per day over a course of 3 days. After an interval of 24 h, two same objects were placed in the testing area for each animal to explore for 10 min before being returned to the cage. After a 1-hour interval, one of the objects was replaced with a new one, and the rat explore the testing area once again but for 3 min. Novel object exploration was registered if the center of the rat's head was oriented within 45° of the new object and within 4 cm of it. Climbing over or sitting on an object was not included. Discrimination time was computed as the time spent at the old object subtracted from the time spent at the novel one.

### Western blot analysis

Protein (30 μg) from the striatum was loaded onto a 10% resolving gel for electrophoresis. Proteins were transblotted onto a polyvinylidene difluoride (PVDF) membrane (Merck Millipore) and then immuno-probed with primary antibodies to rat anti-myelin basic protein (MBP, Abcam, Cambridge, UK, ab7349, 1:1000), rabbit anti-CD86 (Abcam, ab112490, 1:1000), rabbit anti-iNOS (Abcam, ab15323, 1:500), rat anti-Fc RII/III receptor (CD16/32, BD Pharmingen, San Diego, CA, 553142, 1:1000), rabbit anti-C3 (Abcam, ab200999, 1:1000), rabbit anti-ITGAM (Abcam, ab133357, 1:1000), goat anti-Arginase-1 (Santa Cruz Biotechnologies, Dallas, TX, sc-18351, 1:500), mouse anti-C3aR (Santa Cruz Biotechnologies, sc-133172, 1:500), rabbit anti-phospho-STAT3 (Tyr705) (Cell Signaling Technology, Danvers, MA, 9145, 1:1000), rabbit anti-STAT3 (Cell Signaling Technology, 4904, 1:1000), mouse anti-β-actin (Invitrogen, Carlsbad, CA, MA5-15739, 1:1000). The blots were incubated with horseradish peroxidase-conjugated IgG secondary antibody (Hua'an, Hangzhou, China) and then reacted with an enhanced chemiluminescence substrate (Pierce, Rockford, IL). The chemiluminescence results were recorded with an imaging system (Bio-Rad, Hercules, CA).

### Quantitative reverse transcriptase PCR

Total RNA from the striatum was isolated using TRIzol reagent (Invitrogen, #15596026) according to the manufacturer's protocol. Amplification was performed with a fast real-time PCR system (7900HT, ABI, Foster, CA) and a SYBR Premix Ex Taq Kit (TaKaRa, Dalian, China, #RR420L); RNA integrity was verified by electrophoresis on a 2% agarose gel. mRNA levels were normalized to the expression of endogenous control GAPDH in triplicate and were calculated by the 2^-ΔΔCt^ method. The gene-specific primer sequences used were as follows: *Cd86* (FW, AAGACATGTGTAACCTGCACCA; RV, TACGAGCTCACTCGGGCTTA), *Cd16* (FW, AGGGATCATTGGACGCAACA; RV, GACTCCTCTGCACCGAGAAA), *Inos* (FW, CACAGTGTCGCTGGTTTGAA; RV, TCTCCGTGGGGCTTGTAGT),* Il-6* (FW, GGTTTGCCGAGTAGACCTCA; RV, TACCCCAACTTCCAATGCTC), *Tnf-α* (FW, AGTCTGCACAGTTCCCCAAC; RV, TTAGGAAGACACGGGTTCCA), *Il-1β* (FW, TGATCGGTCCCAACAAGGAG; RV, TCCGCTTGGTGGTTTGCTAC), *C1qa* (FW, GCACTGTGCTTCAATTGCAACGAG; RV, GAAGATGCTGTCGGCTTCAGTACC), *C1qb* (FW, GTTCTCACCTTCTGCGACTATGCC; RV, GTGAACAACCTCTTCCTGCTCCAG), *C4b* (FW, ATGATATGCCAGCCGCAGATGAC; RV, CAGGTTACCGTTCCGCCAGATG), *C3* (FW, CGTGCTGATCGAGGATGGTT; RV, ACTTCCCCACTAGGGCTTCT), *C3ar1* (FW, AGGCAATGGGCTGGTGCTGT; RV, CAGGAAGACACTGGCAAACAT), *Itgam* (FW, GACTCCGCATTTGCCCTACT; RV, TGCCCACAATGAGTGGTACAG), *Gapdh* (FW, GATGGTGAAGGTCGGTGTGA; RV, TGAACTTGCCGTGGGTAGAG).

### Tissue immunostaining

30-µm-thick free-floating brain sections were blocked by 10% fetal horse serum for 1 h. The sections were incubated in primary antibody solutions overnight at 4 °C. Rat anti-MBP (Abcam, ab7349, 1:400), mouse anti-SMI32 (Biolegend, San Diego, CA, 801701, 1:100), rabbit anti-APC (Abcam, ab72040, 1:50), rabbit anti-Iba-1 (Wako, Richmond, VA, 019-19741, 1:300), rabbit anti-CD86 (Abcam, ab112490, 1:200), mouse anti-CD68 (AbD Serotec, MCA341, 1:200), rabbit anti-C3 (Abcam, ab200999, 1:200), mouse anti-ITGAM (AbD Serotec, Oxford, UK, MCA618, 1:100), rat anti-C3aR (Hycult Biotech, Uden, Netherlands, HM1123, 1:100) antibodies were used. The sections were then incubated with secondary antibodies (Invitrogen, 1:200) at room temperature for 1 h before imaging.

### CLARITY

Rat brain clearing procedures were performed according to an optimized CLARITY protocol 34, 35. For immunostaining, the 500-μm-thick brain slices were incubated with Rat anti-MBP (Abcam, ab7349, 1:200) and rabbit anti-Iba-1 primary antibodies (Wako, 019-19741, 1:200) for 3 days at 37 °C with shaking. The samples were then incubated with secondary antibodies (Invitrogen, 1:200) at 37 °C for an additional 2 days. Subsequently, the samples were incubated in refractive index matching solution (RIMS, 88% HistodenZ, Sigma-Aldrich, St. Louis, MO, #D2158) for 1 h at room temperature before sample mounting. The samples were protected from light during all CLARITY steps.

### Image acquisition and processing

The 30-µm-thick free-floating brain section and 500-μm-thick clarified rat brain slice samples were imaged using a Nikon A1RMP confocal laser scanning microscope (Nikon Instruments Inc., Tokyo) equipped with a 25× water-immersion objective (Nikon CFI Apo NIR, numerical aperture = 1.0, working distance = 2.8 mm). For CLARITY samples, the imaging volume was 504 μm×504 μm×440 μm with a voxel size of 1.01 μm×1.01 μm×1.00 μm. NIS-Elements AR (Nikon Instruments Inc., Tokyo) was used to create three-dimensional volume renderings for myelin and microglia. The image resolution was 512×512. All images were acquired and processed by a researcher blinded to the experiment design.

### Quantitative analysis

The quantification of immunostaining positive cells in the striatum was performed and data were presented as the number of positive cells and percent stained area per field, respectively. The quantification of SMI32/MBP ratio in the striatum was processed by ImageJ and fluorescence intensity in each field was quantified. The quantification of the distribution of microglia (Iba-1^+^) around myelin sheaths (MBP^+^) was performed by counting the number of microglia cell bodies that touched and localized within each myelin (MBP^+^) in the striatum. The ratio of microglia in contact with myelin relative to the amount of myelin in each image field was calculated. This accounted for the difference in the number of myelin fragments in different image fields and was considered to represent changes in the redistribution pattern of microglia in relation to myelin. For the quantification of the distribution of CD86^+^ microglia around each myelin fiber (MBP^+^), a region of interest (ROI) was drawn encompassing two concentric circles starting from the diameter of each myelin and ending at a 15-μm ascending radius. Threshold was set and the area (μm^2^) of CD86^+^ puncta within each ROI was quantified. For the quantification of the deposition of C3 puncta on each myelin fiber (MBP^+^), a region of interest encompassing each myelin within the striatum was drawn. Threshold was set and the area (μm^2^) of C3^+^ puncta within each myelin was quantified. For each rat, at least 4 images at 25× magnification were counted, which were derived from 4 fixed-frozen coronal sections spaced 100 μm apart. All quantifications were performed in NIS-Elements AR analysis software (Nikon Instruments Inc., Tokyo). Quantitative analyses of CLARITY images were performed using our customized MATLAB code. The procedures were described previously 35. For each animal, at least 6 regions of interest in the striatum at 25× magnification were counted. The percent of microglia in contact with myelin relative to the amount of myelin in the 3D volume was calculated. Image quantification and analyses were performed by researchers blinded to group assignments.

### Statistical analysis

Descriptive statistics are presented as the mean ± standard deviation (SD). The normal distribution of data was examined by the Kolmogorov-Smirnov test. Results from Morris water maze spatial learning tests were compared by repeated measures two-way ANOVA. For all other comparisons among different groups, Student's t-test or one-way ANOVA followed by Newman-Keuls* post hoc* test (homogeneity of variance) or Dunnett's *post hoc* test (heterogeneity of variance) were used. For nonparametric analysis, Mann-Whitney U-test and Kruskal-Wallis test followed by Dunnett's* post hoc* analysis were used. Statistical analyses were performed using SPSS software (version 17.0, SPSS, Chicago, IL). Two-tailed *P* values less than 0.05 were considered statistically significant.

## Results

### Cerebral vessel density and dynamic cerebral blood flow decreased after BCCAO

Synchrotron radiation angiography (SRA) indicated the formation of collaterals at day 28 after BCCAO (**Figure 1B**). This included the internal carotid artery to the ophthalmic artery and the internal carotid artery to the pterygopalatine artery. The quantitative results showed that the diameters of the anterior cerebral artery, middle cerebral artery, and posterior cerebral artery increased from day 7 to day 28 after BCCAO (*p*< 0.0001 for the anterior cerebral artery; *p*= 0.001 for the middle cerebral artery; *p*< 0.0001 for the posterior cerebral artery, **Figure 1C**). Compared with those in the control group, the densities of the cerebral vessels decreased after BCCAO (*p*< 0.0001, **Figure 1C**).

Three-dimensional arterial spin labeling was used to monitor the dynamic changes in cerebral blood flow. The cerebral blood flow of the whole brain decreased to 50 ± 9.3% of the baseline level at day 7 after BCCAO (*p*< 0.0001) and remained at 58 ± 20.4% of the baseline level at day 14 after BCCAO (*p*= 0.002). At day 28 after BCCAO, the cerebral blood flow of the whole brain was 112 ± 26.5% of the baseline level, which may be due to collateral opening (*p*= 0.750, **Figure 1D**). Furthermore, we found that cerebral blood flow changes in the cortex and striatum were consistent with those in the whole brain.

### BCCAO induced spatial learning and memory impairments and white matter injury

The Morris water maze test was used to evaluate spatial learning and memory performance in BCCAO and control rats. The quantitative data showed that the latency to the platform was longer at day 14 and day 28 after BCCAO compared with the controls in the spatial acquisition test (*p*= 0.020 for day 14 and *p*= 0.002 for day 28, **Figure S1A**). We also found that the percentage time in the target quadrant was lower at day 28 (*p*= 0.049) but not day 14 (*p*= 0.839) after BCCAO compared to the controls in the probe test (**Figure S1B**). However, no differences were found in spatial learning or memory performance between BCCAO and control rats at day 7 after surgery (*p*= 0.160 for spatial learning test and *p*= 0.813 for spatial memory test, **Figure S1A** and **S1B**). The data indicated that BCCAO rats exhibited spatial learning and memory deficits beginning at day 14 and lasting to day 28. Furthermore, working memory was evaluated in BCCAO and controls rats after 28 days of surgery. The latency to the platform was longer on day 1 of the test in BCCAO rats compared to the controls (*p*= 0.022, **Figure S4B**), which suggested that chronic cerebral hypoperfusion also induced working memory impairment.

Since cognitive dysfunction in chronic ischemia is associated with white matter injury 4, 9, we investigated the expression of the myelin marker myelin basic protein in BCCAO and control rats. The quantitative results showed that MBP protein levels in the striatum were lower in BCCAO rats than in control rats from day 7 to day 28 (*p*= 0.0002, **Figure S1C**). Moreover, the fluorescence intensity of SMI32 increased and APC decreased in the striatum of BCCAO rats compared to the control rats (*p*= 0.013 for SMI32; *p*< 0.001 for APC, **Figure S1D**), which indicated that BCCAO induced axonal and oligodendrocyte damage. In addition, quantification of MBP immunostaining in five additional tested white matter regions showed consistent myelin degradation after hypoperfusion (*p*< 0.05 for all tested region, **Figure S2**). These data demonstrated that BCCAO induced white matter injury starting at day 7 and sustained for at least 28 days.

### Increased reactive microglia and neuroinflammation in BCCAO rats

**Figure 2:**
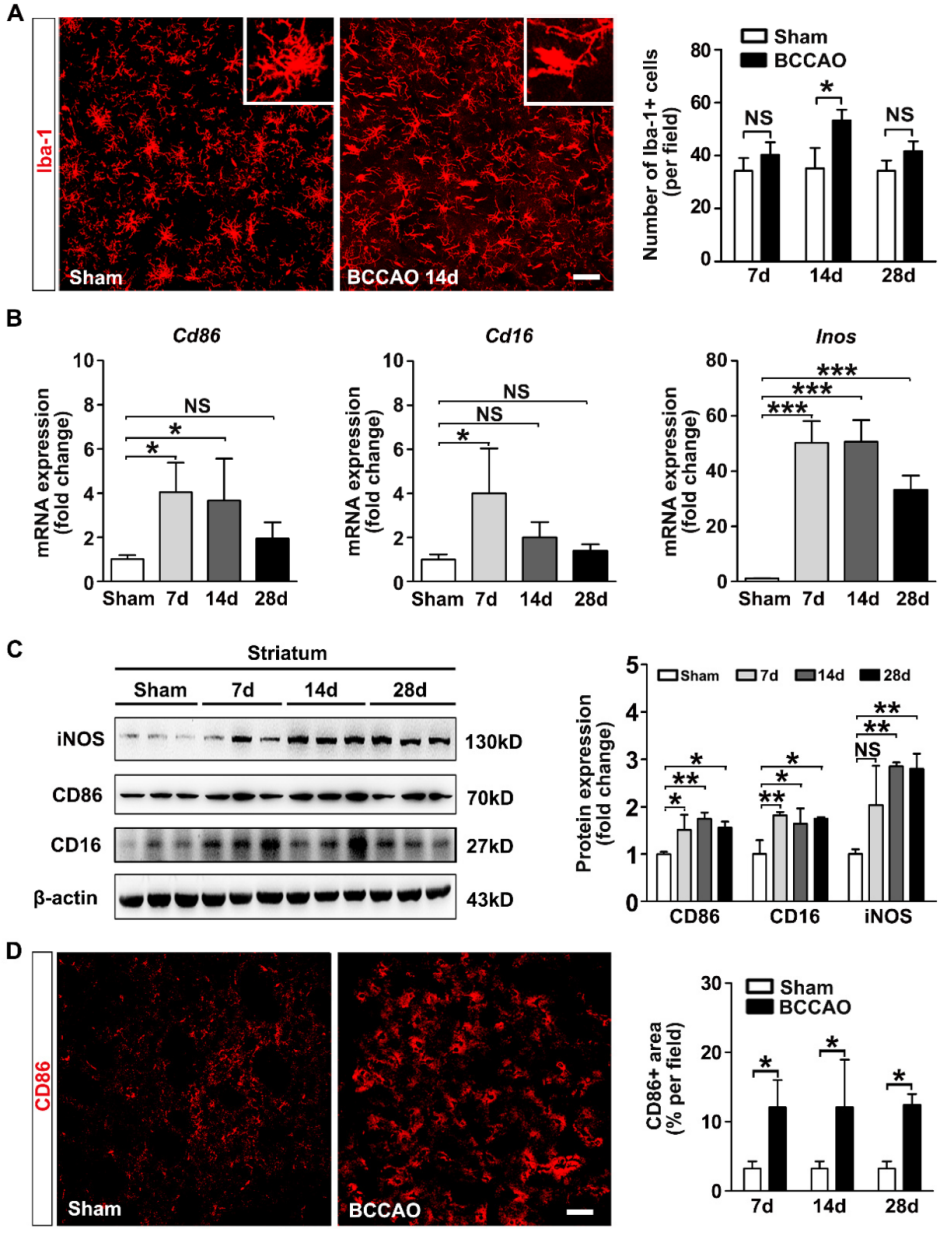
** Aberrant activated microglia increase in BCCAO rats. A** Representative images and quantification for the number of Iba-1^+^ microglia cells (red) in the striatum of control and BCCAO rats at day 7, 14, and 28 after surgery. n = 3-4 animals in each group. Scale bar=50 μm. **B** Quantitative RT-PCR analysis of the expression of *Cd86*,* Cd16*, and *Inos* in the striatum of control and BCCAO rats at day 7, 14, and 28 after surgery. The values are normalized to those of the control group. n = 3-6 in each group. **C** Western blots and quantification for CD86, CD16, iNOS, and β-actin in the striatum of BCCAO and control rats at day 7, 14, and 28 after surgery. n = 3 in each group. **D** Representative images and quantification of reactive microglia (CD86^+^ cells, red) in the striatum of BCCAO and control rats. n = 3-4 animals in each group. Scale bar=50 μm. The data are shown as the mean ± SD. ***, *p*< 0.001; **, *p*< 0.01; *, *p*< 0.05; NS, not significant; the BCCAO group vs. control group.

We further evaluated whether microglia are activated after surgery. We found that Iba-1^+^ cells in the striatum exhibited small soma and ramified processes before surgery. After surgery, microglia displayed enlarged cell bodies with rarefied and shortened processes, which are characteristic morphology of activated microglia (**Figure 2A**). The number of Iba-1^+^ cells in the striatum was increased at day 14 after surgery compared to the controls (*p*= 0.014, **Figure 2A**). The mRNA and protein expression of pro-inflammatory microglia markers CD86, CD16, and iNOS in the striatum were up-regulated in BCCAO rats compared with that in control rats (*p*= 0.015 for *Cd86* mRNA; *p*= 0.013 for* Cd16* mRNA;* p*< 0.001 for *Inos* mRNA;* p*= 0.006 for CD86 protein; *p*= 0.007 for CD16 protein;* p*= 0.003 for iNOS protein, **Figure 2B** and **2C**). The mRNA levels of the pro-inflammatory cytokines IL-6, TNF-α, and IL-1β in the striatum were also significantly increased in BCCAO rats compared to those in control rats (*p*= 0.001 for *Il-6*; *p*= 0.001 for *Tnf-α*; *p*= 0.001 for *Il-1β*, **Figure S3A**). Furthermore, the number of CD86^+^ cells was increased in BCCAO rats (*p*= 0.026, **Figure 2D**). But no significant difference was found in the protein levels of the anti-inflammatory marker Arginase-1 in the striatum between BCCAO and control rats (*p*= 0.203, **Figure S3B**). These data suggested that microglia activated towards detrimental phenotype and induced neuroinflammation after hypoperfusion.

### Reactive microglia redistributed and phagocytosed myelin in the striatum of BCCAO rats

**Figure 3:**
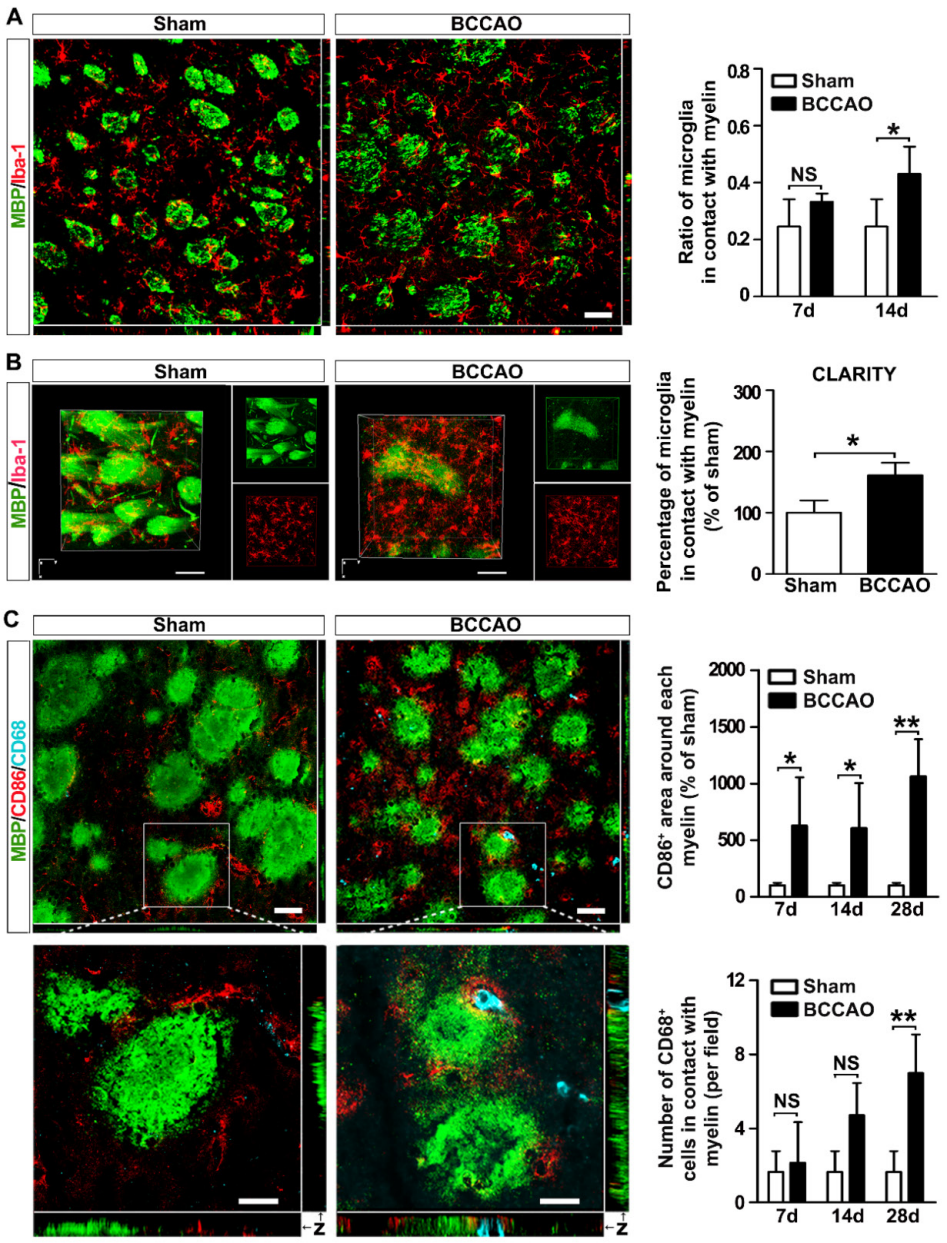
** Microglia redistribute and phagocytose myelin in the striatum of BCCAO rats. A** Representative images and quantification of the proportion of microglia cells (Iba-1^+^ cells, red) adhered to myelin (MBP^+^, green) relative to the number of myelin fibers in the striatum of control and BCCAO rats. Scale bar=50 μm.** B** CLARITY analysis of 500-μm-thick rat brain slice co-stained with Iba-1 and MBP in control and BCCAO rat at day 14 after surgery. The volumes of representative 3D visualization are 309 μm×309 μm×200 μm, with a voxel size of 1.01 μm×1.01 μm×1.00 μm. Scale bar=100 μm. **C** Triple immunostaining and quantification of reactive (CD86^+^ cells, red) and phagocytic microglia cells (CD68^+^ cells, indigo) adhered to myelin (MBP^+^, green) in the striatum of control and BCCAO rats at day 7, 14, and 28 after surgery. Images at the bottom are magnified views of the areas in the upper of the images. Scale bar=50 μm for images at the top, Scale bar=10 μm for images at the bottom. n = 3-4 animals in each group. The data are shown as the mean ± SD. **, *p*< 0.01; *, *p*< 0.05; NS, not significant; the BCCAO group vs. control group.

Chemotaxis and phagocytosis are two major functions of microglia. We investigated whether these functional behaviors of microglia were involved in ischemic white matter injury. Quantification of the microglia proportion in contact with the myelin fibers showed an increasing tendency from day 7 to day 14 after BCCAO surgery compared to controls (*p*= 0.012,** Figure 3A**), suggesting microglia moved towards myelin after BCCAO surgery. Furthermore, to determine the spatial distribution of microglia relative to myelin in the striatum, the CLARITY imaging method was used. At day 14 after surgery, compared with control rats, BCCAO rats displayed an uneven distribution of microglia, and more microglia were in contact with the myelin fibers in the striatum (*p*= 0.021, **Figure 3B** and** Suppl. Movie 1**).

To further analyze the phenotype of the microglia adhering to myelin and their role, we used triple-staining of MBP with the pro-inflammatory marker CD86 and the lysosomal proteins CD68 in BCCAO and control rats (**Figure 3C**). The results showed significantly increased levels of CD86 immunoreactivity around each myelin after BCCAO surgery (*p*= 0.011, **Figure 3C**). Furthermore, phagocytotic microglia (CD68^+^) in contact with myelin also tended to increase after BCCAO surgery (*p*= 0.023, **Figure 3C**). The colocalization of CD68 and CD86 suggested that phagocytotic activity of the reactive microglia increased after BCCAO surgery (**Figure 3C**) and accompanied by decreased myelin fibers in the striatum. Taken together, these results suggested that aberrant activated microglia redistributed and phagocytosed myelin to aggravate white matter injury.

### Activation of the C3-C3aR/ITGAM pathway in BCCAO rats

**Figure 4:**
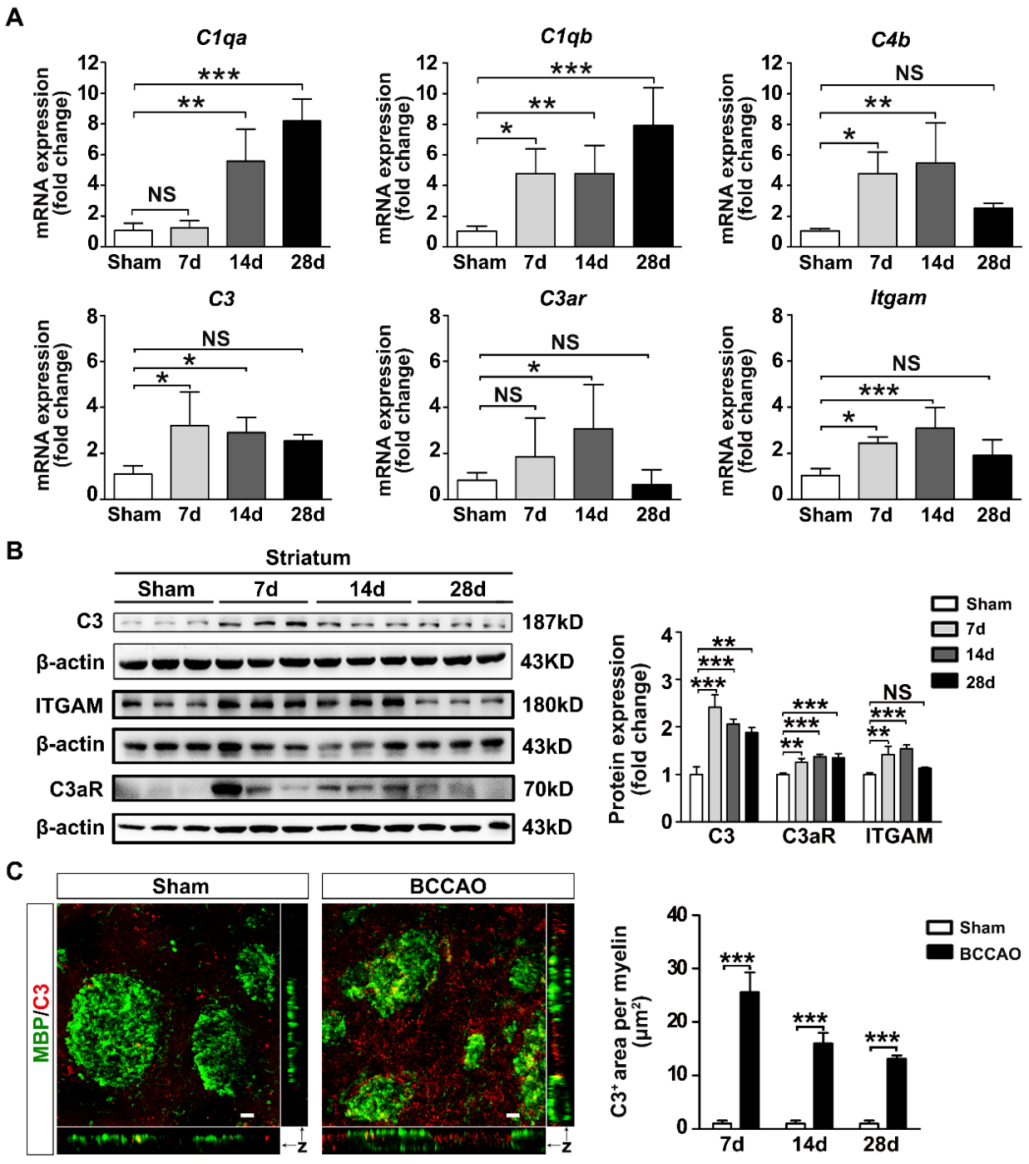
** Activation of complement C3-C3aR/ITGAM pathway in BCCAO rats. A** Quantitative RT-PCR analysis of the expression of* C1qa, C1qb, C4b, C3*,* C3ar*, and *Itgam* in the striatum of control and BCCAO rats at day 7, 14, and 28 after BCCAO surgery. The values are normalized to those of the control group. n = 3-7 in each group. **B** Western blots and quantification for C3, C3aR, ITGAM, and β-actin in the striatum of control and BCCAO rats at day 7, 14, and 28 after surgery. **C** Representative images and quantification of complement C3 puncta (red) deposition on myelin (MBP^+^, green) in the striatum of BCCAO and control rats. n = 3-4 animals in each group at day 7, 14, and 28 after surgery. Scale bar=10 μm. The data are shown as the mean ± SD. ***, *p*< 0.001; **, *p*< 0.01; *, *p*< 0.05; NS, not significant; the BCCAO group vs. the control group.

Microglias express high levels of ITGAM (also known as CR3 or CD11b) immunoreactivity which are up-regulated under chronic cerebral ischemia 12. Since ITGAM is one of the receptors of complement component C3, we determined whether the C3-C3aR/ITGAM signaling pathway was involved in white matter injury after BCCAO injury. The mRNA and protein levels of complement C3 and its receptors C3aR and ITGAM in BCCAO rats were examined. In addition, the mRNA levels of major complement components C1qa, C1qb, C4b were also detected. The results showed that the mRNA levels of complement components C1qa, C1qb, C3, C4b were increased in BCCAO rats (*p*< 0.0001 for *C1qa* mRNA comparison; *p*= 0.002 for *C1qb* mRNA comparison; *p*= 0.012 for *C3* mRNA comparison; *p*= 0.005 for *C4b* mRNA comparison; **Figure 4A**). C3 protein levels in the striatum were also increased in BCCAO rats compared with those in control rats (*p*< 0.0001,** Figure 4B**). Moreover, the mRNA and protein levels of ITGAM in the striatum were increased in BCCAO rats at day 7 and day 14 after surgery compared to those in control rats (*p*= 0.001 for mRNA comparison; *p*= 0.0005 for protein comparison, **Figure 4A** and** 4B**). Importantly, we found that C3 deposition on myelin fibers dramatically increased after BCCAO surgery (*p*< 0.0001, **Figure 4C**). At the same time, the mRNA and protein levels of C3aR, another receptor of complement C3, were significantly up-regulated after BCCAO surgery (*p* = 0.021 for mRNA comparison;* p*= 0.0005 for protein comparison, **Figure 4A** and** 4B**). These results indicated that the C3-C3aR/ITGAM pathway was activated after BCCAO injury.

### *C3ar1* ablation alleviated microglia activation and white matter impairment in BCAS mice

**Figure 5:**
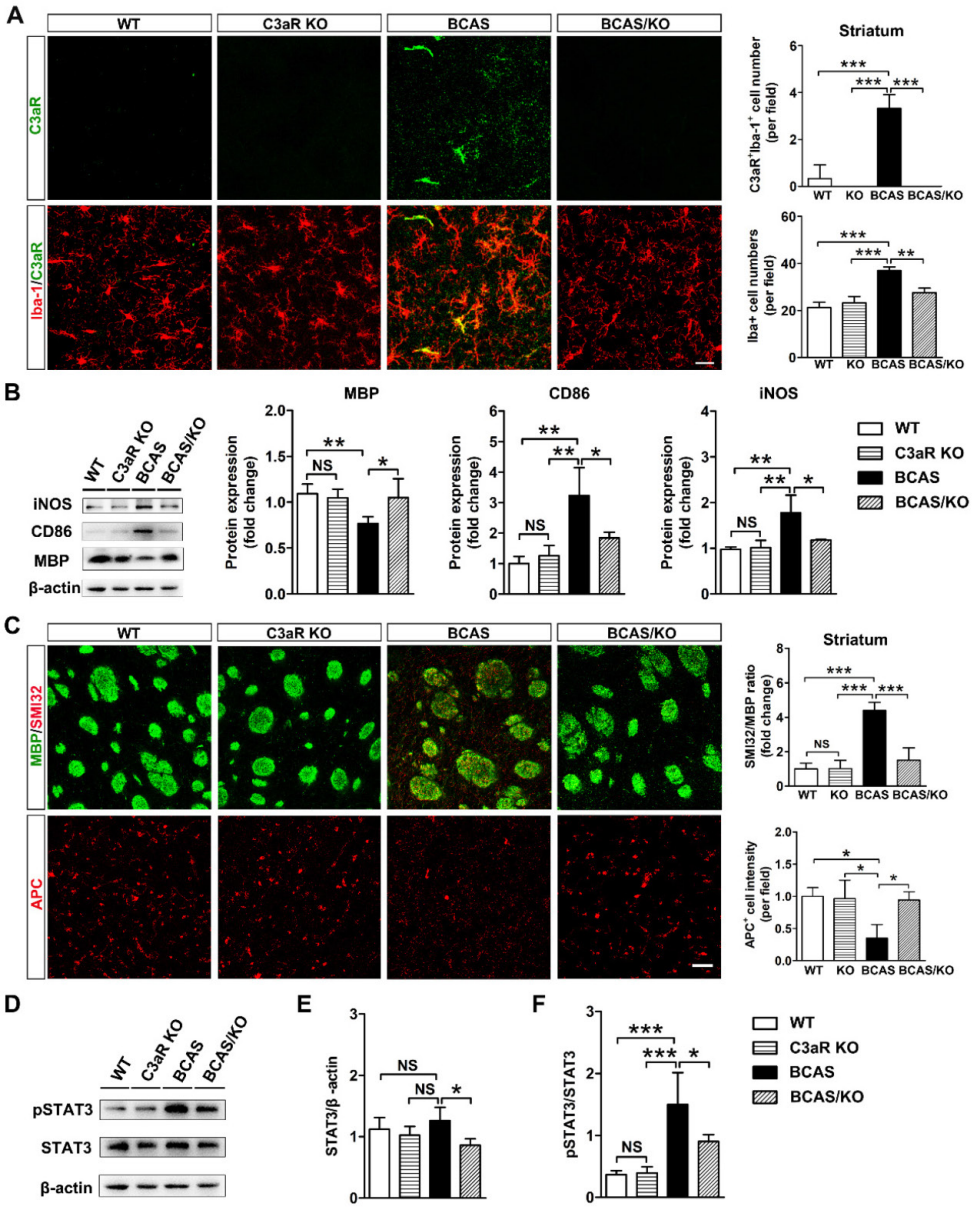
** Genetic deletion of *C3ar1* attenuates microglia activation and reverses white matter injury in BCAS mice. A** Representative images and quantification of C3aR (green) and Iba-1 (red) double-positive cells and microglia cells (Iba-1^+^ cells, red) in the striatum of WT, C3aR-KO, BCAS, and BCAS/C3aR-KO mice at day 28 after surgery. Scale bar=25 μm.** B** Western blots and quantification for CD86, iNOS, MBP and β-actin in the striatum of WT, C3aR-KO, BCAS, and BCAS/C3aR-KO mice at day 28 after surgery. **C** Representative images and quantification of damaged axon (SMI32^+^, red) relative to myelin (MBP^+^, green) and mature oligodendrocyte (APC^+^ cells, red) in the striatum of WT, C3aR-KO, BCAS, and BCAS/C3aR-KO mice at day 28 after surgery. Scale bar=50 μm.** D** Western blots of total- and phospho-STAT3 (pSTAT3) and β-actin in the striatum of WT, C3aR-KO, BCAS, and BCAS/C3aR-KO mice at day 28 after surgery. **E** and **F** Quantification of total STAT3/ β-actin (**E**) and phospho-STAT3/STAT3 (**F**) levels in the striatum of WT, C3aR-KO, BCAS, and BCAS/C3aR-KO mice at day 28 after surgery. The data are shown as the mean ± SD. n = 3-4 animals in each genotype group. ***, *p*< 0.001; **, *p*< 0.01; *, *p*< 0.05; NS, not significant.

Complement component C3 must be split into its cleavage products C3a and iC3b to execute its biological function 36. We hypothesized that the up-regulation of C3 would mediate microglial activation and chemotaxis towards myelin fibers by binding C3aR on microglia membrane. Subsequently, microglia opsonize myelin fragments for elimination through ITGAM. C3aR/Iba-1 double-staining revealed predominantly increases C3aR fluorescence in Iba-1-positive area in BCAS mice (*p*< 0.0001, **Figure 5A**). Then C3aR knockout mice were used to evaluate C3-C3aR signaling on microglia activation and white matter injury after hypoperfusion. We found that the number of microglia was significantly decreased in C3aR knockout mice compared with those wild type after BCAS surgery (*p*< 0.001, **Figure 5B**). Genetic deletion of C3aR significantly down-regulated the expression of reactive microglial marker CD86 and iNOS protein levels in the striatum after BCAS surgery (*p*= 0.001 for CD86; *p*= 0.001 for iNOS, **Figure 5B**). MBP protein levels were also recovered in the striatum of C3aR knockout mice after BCAS surgery (*p*= 0.007, **Figure 5B**). SMI32 and APC immunostaining results showed that C3aR ablation significantly rescued axon and oligodendrocyte in BCAS mice at day 28 after surgery (*p*= 0.0001 for SMI32; *p*= 0.012 for APC*,* **Figures 5C**). Moreover, deleting microglia by CSF1R inhibitor PLX3397 or inhibiting microglia activation by minocycline, axonal and oligodendrocyte degeneration were significantly reversed induced by chronic cerebral hypoperfusion (*p*< 0.01 for all comparisons, **Figure S6** and **S7**). The results indicated that C3-C3aR pathway directly mediated aberrant microglia activation and white matter injury in chronic cerebral hypoperfusion.

It is reported that STAT3 was a downstream target of C3-C3aR signaling pathway 37. We detected phospho-STAT3 and STAT3 protein levels in our chronic hypoperfusion model. We found that genetic deletion of C3aR significantly inhibited STAT3 phosphorylation in the striatum at day 28 after surgery (*p*= 0.0004, **Figure 5D**). The results suggested that C3aR mediated microglia activation via STAT3 signaling.

### C3aR inhibition prevents microglial activation and redistribution after BCCAO

**Figure 6:**
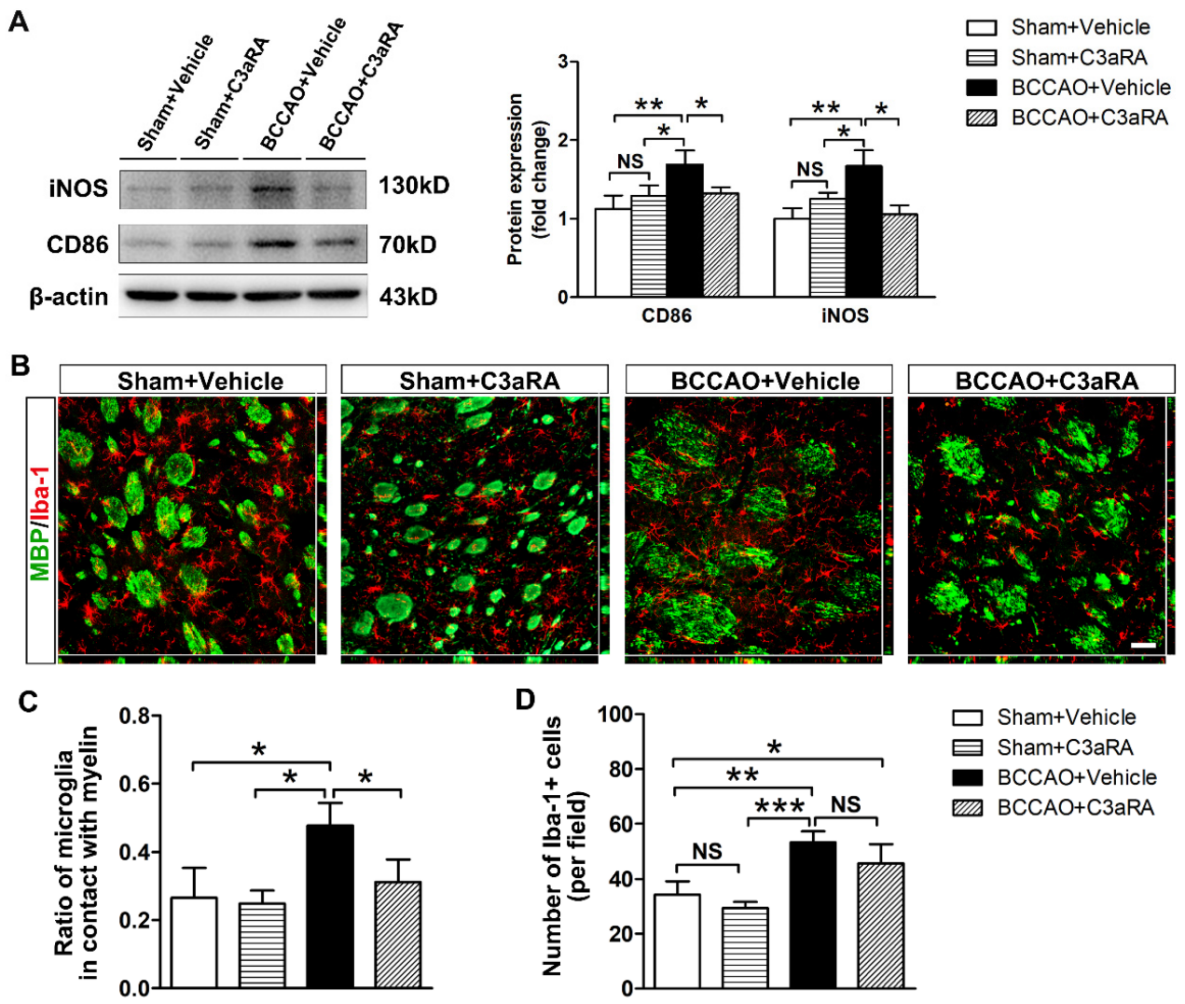
** C3aR inhibition suppresses microglial activation and microglia redistribution to myelin in BCCAO rats. A** Western blots and quantification for CD86, iNOS, and β-actin in the striatum of the sham+vehicle, sham+C3aR antagonist, BCCAO+vehicle, and BCCAO+C3aR antagonist groups. n = 3-6 in each group. **B** Representative images of microglia cells (Iba-1^+^ cells, red) contacting with myelin (MBP^+^, green) in the striatum of sham+vehicle, sham+C3aR antagonist, BCCAO+vehicle, and BCCAO+C3aR antagonist groups. Scare bar=50 μm. n= 3-4 in each group. **C-D** Quantification of the proportion of microglia cells adhered to myelin relative to the number of myelin fibers (**C**) and the number of microglia cells (**D**). The data are shown as the mean ± SD. ***, *p*< 0.001; **, *p*< 0.01; *, *p*< 0.05; NS, not significant; the BCCAO group vs. control group. C3aRA: C3aR antagonist.

SB290157, a specific antagonist of C3aR, was used to further evaluate the role of C3-C3aR signaling in mediating microglia activation and chemotaxis. Firstly, we monitored the dynamic CBF changes under C3aR inhibition in BCCAO and the control rats by using three-dimensional arterial spin labeling technique. The quantitative results showed that CBF of the whole brain decreased to 68.38 ± 11.27% of the baseline level at day 7 after BCCAO under C3aR inhibition (*p*< 0.05) and remained at 68.37 ± 15.06% of the baseline level at day 14 after BCCAO surgery under C3aR inhibition (*p*< 0.05). At day 28 after BCCAO surgery, CBF of the whole brain under C3aR inhibition was 72.07 ± 12.39% of the baseline (*p*< 0.05, **Figure S5**). No significant difference was found in CBF changes from day 7 to 28 between BCCAO rats with and without C3aR inhibition. We then determined the protein expression of reactive microglia markers CD86 and iNOS in the striatum of BCCAO rats treated intraperitoneally with SB290157 or vehicle. The results showed that compared with vehicle injection, SB290157 treatment inhibited CD86 and iNOS protein expression after BCCAO surgery (*p*= 0.002 for CD86; *p*= 0.002 for iNOS, **Figure 6A**).

We further performed MBP/Iba-1 co-staining to evaluate whether C3aR inhibition affected microglia mobility towards myelin in BCCAO rats. Iba-1 immunostaining showed that microglia exhibited shorter cellular processes without enlarged cell bodies in BCCAO rats treated with SB290157 compared with those treated with vehicle (**Figure 6B**), indicating that C3aR inhibition weakened the morphological change of microglia after hypoperfusion. Furthermore, compared with vehicle treatment, SB290157 treatment significantly decreased the proportion of microglia in contact with myelin in BCCAO rats (*p*= 0.008, **Figure 6C**), suggesting that blocking C3aR pathway inhibited microglial movement towards myelin. However, SB290157 treatment did not significantly reduce the number of microglia in the striatum in comparison with vehicle treatment (*p*= 0.158, **Figure 6D**). These results indicated that targeting C3aR prevented microglia activation and redistribution in the striatum after BCCAO.

### C3aR inhibition prevents behavioral deficits and white matter injury in BCCAO rats

**Figure 7:**
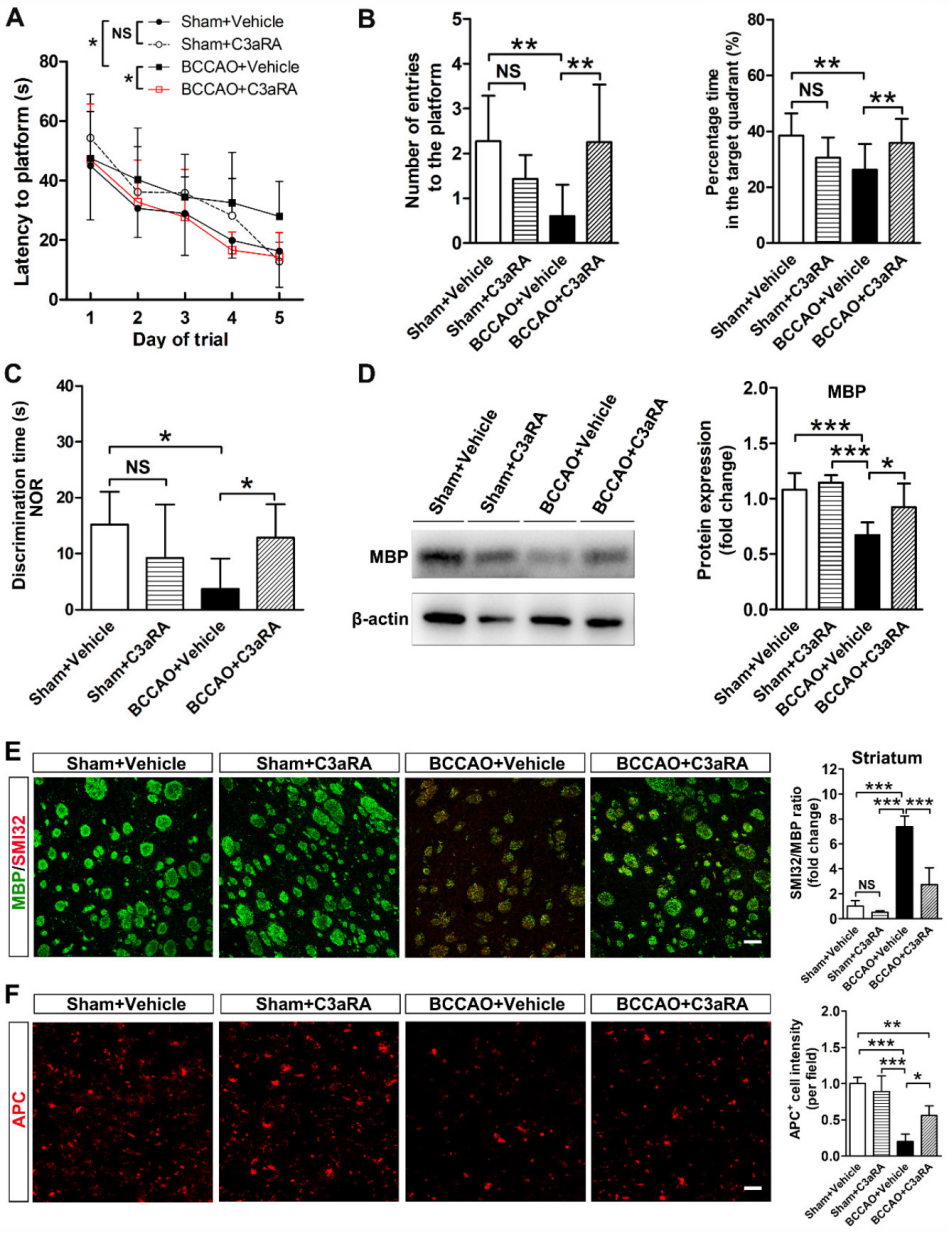
** C3aR inhibition prevents behavioral deficits and white matter injury in BCCAO rats. A** Five-day spatial learning performance measured as the latency to reach the platform in the Morris water maze test in sham+vehicle, sham+C3aR antagonist, BCCAO+vehicle, and BCCAO+C3aR antagonist groups. n= 12-15 animals for each group. **B** Spatial memory performance measured as the number of entries into the platform quadrant and the percentage of time spent in the platform quadrant in the Morris water maze test in the sham+vehicle, sham+C3aR antagonist, BCCAO+vehicle, and BCCAO+C3aR antagonist groups, n = 12-15 animals in each group. **C** Spatial memory performance measured as discrimination time in the new object recognition test in the sham+vehicle, sham+C3aR antagonist, BCCAO+vehicle, and BCCAO+C3aR antagonist groups. n = 4-6 animals for each group. **D** Western blots and quantification for myelin basic protein (MBP) and β-actin in the striatum of sham+vehicle, sham+C3aR antagonist, BCCAO+vehicle, and BCCAO+C3aR antagonist groups. **E**-**F** Representative images and quantification of damaged axon (SMI32^+^, red) relative to myelin (MBP^+^, green) (**E**) and mature oligodendrocyte (APC^+^ cells, red) (**F**) in the striatum of sham+vehicle, sham+C3aR antagonist, BCCAO+vehicle, and BCCAO+C3aR antagonist groups. n = 3-4 animals in each group. Scale bar=50 μm. The data are shown as the mean ± SD. **, *p*< 0.01; *, *p*< 0.05; the BCCAO group vs. control group. C3aRA: C3aR antagonist.

To further determine whether SB290157 could alleviate cognitive impairment after BCCAO, Morris water maze and new object recognition tests were performed. Spatial learning and memory dysfunction were reversed by SB290157 treatment in BCCAO rats in the Morris water maze test (*p*= 0.002 for escape latency to the platform; *p*= 0.010 for the number of entries onto the platform; *p*= 0.006 for percentage time in the target quadrant,** Figures 7A** and** 7B**). SB290157 treatment also led to a significant recovery of cognitive function after BCCAO in the new object recognition test (*p*= 0.013, **Figure 7C**). The results indicated that C3aR antagonist SB290157 could effectively alleviate cognitive impairment due to BCCAO surgery.

Furthermore, MBP protein levels were recovered in BCCAO rats treated with SB290157 (*p*= 0.0002, **Figure 7D**). SMI32 and APC immunostaining results showed that SB290157 treatment significantly alleviated axonal and oligodendrocyte degeneration in BCCAO rats than that in controls after 28 days of surgery (*p*< 0.0001 for SMI32; *p*< 0.0001 for APC, **Figure 7E** and **7F**). These results indicated recovery of white matter integrity after SB290157 treatment in BCCAO rats.

## Discussion

In the present study, we combined SRA with three-dimensional arterial spin labeling to detect cerebral vessel density and blood flow after BCCAO injury for the first time. Microglia were activated, which moved and adhered to myelin and phagocytosed myelin components to aggravate white matter injury via C3-C3aR pathway after chronic hypoperfusion. Furthermore, genetic deletion of *C3ar1* and C3aR antagonist inhibited aberrant microglia activation and microglia redistribution to myelin and subsequently reversed white matter injury and cognitive deficits.

Microglia can be activated by different signals in the micromilieu to trigger a pro-inflammatory or anti-inflammatory response, thus exacerbating tissue damage or promoting tissue repair 38. In multiple sclerosis, microglia activation is correlated with detrimental clinical outcomes 39. Inhibiting microglia activation to the pro-inflammatory phenotype could alleviate inflammatory injury, limit myelin loss in experimental autoimmune neuritis and chronic cerebral hypoperfusion 40, 41. Our results support that detrimental microglia increase and release pro-inflammatory cytokines after BCCAO. Previous reports suggested that microglial phagocytosis of myelin debris is considered beneficial and a prerequisite for myelin regeneration and axonal growth 42, 43. Our findings support the notion that reactive microglia expressing CD68 adhered to and engulfed the intact myelin fibers rather than the myelin debris. The active phagocytosis of myelin was concomitant with cognitive dysfunction, suggesting that myelin phagocytosis by microglia destroyed white matter integrity after BCCAO.

Another important finding of our study is that the activation of the complement C3-C3aR pathway exacerbated white matter injury through microglia activation and redistribution in chronic cerebral hypoperfusion. Recent study has reported that complement component C5 is involved in white matter injury in chronic cerebral ischemia 25. In addition, a previous study showed that disrupting microglia specific C3/CR3 signaling resulted in sustained deficits in synaptic connectivity 44. Our results indicate that increased C3 levels aggravate white matter injury after hypoperfusion via microglial C3a receptor.

C3aR is expressed on neurons, microglia, astrocytes, endothelial cells, and stem cells, but its expression in neurons was not dramatically up-regulated during neuroinflammation 45. In contrast, its expression in microglia was significantly up-regulated in disease models 37, 45, 46. Inhibiting C3aR reduced neuroinflammation in AD mouse model 37. In addition, C3aR^-/-^ inhibits the activation of microglia, subsequently blocks the phagocytosis of presynaptic components in the hippocampus and reverses the cognitive impairments caused by West Nile virus infection 22. We found that C3aR predominantly increased in Iba-1-positive cells and blocking C3aR pathway suppressed detrimental microglial activation and alleviated white matter injury and cognitive decline after hypoperfusion. Furthermore, treatment with the C3aR antagonist modified microglia morphology and inhibited microglial movement to myelin fibers. However, the present study does not rule out the possibility that up-regulation of C3aR may occasionally occur on neurons, astrocytes, and endothelial cells, the therapeutic effects of C3aR antagonists after hypoperfusion may occur indirectly by modulating the functions of these cell types. Further studies are needed to determine the contribution of these cell types to disease pathogenesis.

In the present study, we found that genetic deletion of C3aR inhibited STAT3 phosphorylation in the striatum after hypoperfusion, suggesting that C3aR regulated microglia activation via STAT3 signaling. This finding was consistent with a previous study reporting that STAT3 was a downstream target of C3-C3aR signaling and C3aR activates STAT3 signaling in microglia of AD transgenic mice through direct and indirect mechanisms 37. Considering the complex crosstalk among various immune pathways, other signaling pathways may work in parallel to augment the microglia activation in downstream of C3aR after hypoperfusion. Further study will be needed to identify their roles in complement C3aR-mediated microglia activation after hypoperfusion.

CLARITY imaging and analysis system provide a powerful tool for thoroughly evaluating spatial structural and functional alteration of brain in rodent disease models. It facilitates antibody penetration into the brain by removing lipid bilayers, and permitted rounds of staining. In the present study, moreover, various fluorescent signal amplification systems were tried to improve antibody penetration throughout the thickness of the sample. We applied longer clearing time before antibody incubation, the higher concentration of antibodies. In addition, higher laser intensity was used to achieve high quality of signal-to-noise ratio in deep imaging of brain section. However, the inherent difficulty of poor antibody penetration into the thick brain sections still exists in this novel clearing technique. CLARITY-optimized whole brain staining method should be developed in future study.

It is worth noting that our analysis mainly focused on the resident microglia in the CNS. Activated Iba-1-positive cells might also be infiltrated macrophages. Further studies using bone marrow (BM)-chimerism and dual-reporter transgenic fate mapping and next-generation sequencing are needed to distinguish the potential effect of infiltrated macrophages from resident microglia.

In conclusion, the up-regulation of complement C3 expression activated the microglial membrane receptors C3aR, which induced aberrant microglia activation and mediated microglia redistribution and myelin engulfment in the striatum during chronic cerebral hypoperfusion. Pharmacological targeting of C3aR alleviated white matter injury and eventually reversed cognitive decline. Given that white matter impairment is a canonical feature of human small-vessel disease and vascular dementia, our study demonstrated a critical role of C3aR in mediating neuroinflammation and white matter injury through aberrant microglia activation, which provides a novel therapeutic strategy for the small-vessel disease and vascular dementia.

## Supplementary Material

Supplementary figures and movie legend.

Supplementary movie.

## Abbreviations

AD: Alzheimer's disease
BCCAO: bilateral common carotid artery occlusion
BCAS: bilateral common carotid artery stenosis
C3aR: C3a receptor
C3aRA: C3a receptor antagonist
CBF: cerebral blood flow
CCD: charge coupled device
CNS: central nervous system
SRA: synchrotron radiation angiography
