# Supplementary material for: Microglia exacerbate white matter injury via complement C3/C3aR pathway after hypoperfusion
Source: Theranostics. 2020 Jan 1;10(1):74–90. doi: 10.7150/thno.35841 (PMC6929610; doi:10.7150/thno.35841)
Supplement: Supplementary file 1 — Supplementary figures and movie legend. [file thnov10p0074s1.pdf]

Supplemental Figures and Figure Legends

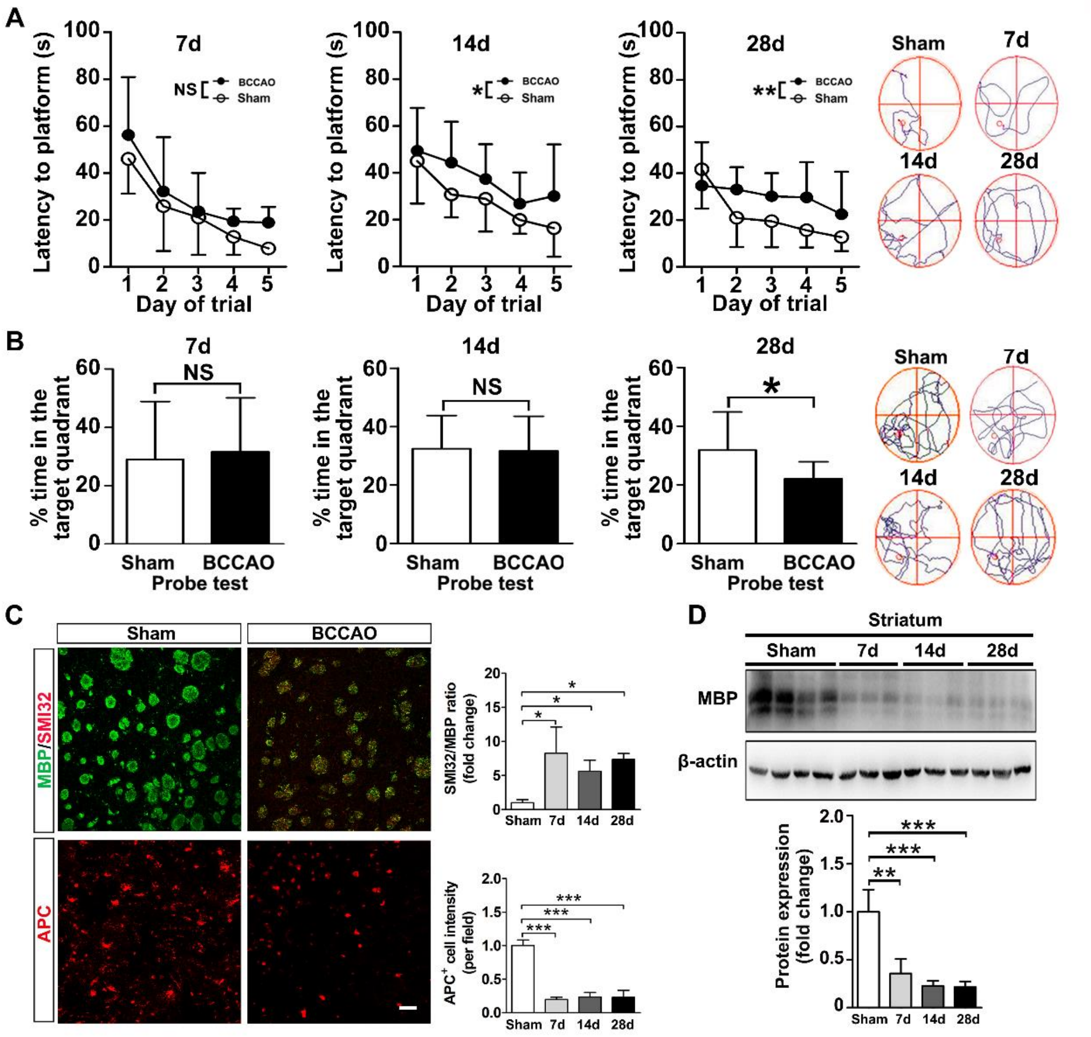

Figure S1 BCCAO-induced spatial learning and memory impairments and myelin degradation. A Five-day spatial learning performance measured as the latency to reach the platform in the Morris water maze test and representative swimming paths in control and BCCAO rats at day 7, 14, and 28 after surgery. n = 12-15 animals in each group. **B** Spatial memory performance measured as the percentage of time spent in the platform quadrant in the Morris water maze test and representative swimming paths for control and BCCAO rats at day 7, 14, and 28 after

surgery. n = 12-15 animals in each group. **C** Representative images and quantification of damaged axon ($SMI32^+$, red) relative to myelin (MBP^+, green) and mature oligodendrocyte (APC^+ cells, red) in the striatum of BCCAO and control rats. n = 3-4 animals in each group. Scale bar=50 μm. **D** Western blots and quantification for MBP and β-actin in the striatum of control and BCCAO rats at day 7, 14, and 28 after surgery. n=3 in each group. The data are shown as the mean ± SD. ***, $p< 0.001$; **, $p< 0.01$; *, $p< 0.05$; NS, not significant; the BCCAO group vs. control group. MBP: myelin basic protein.

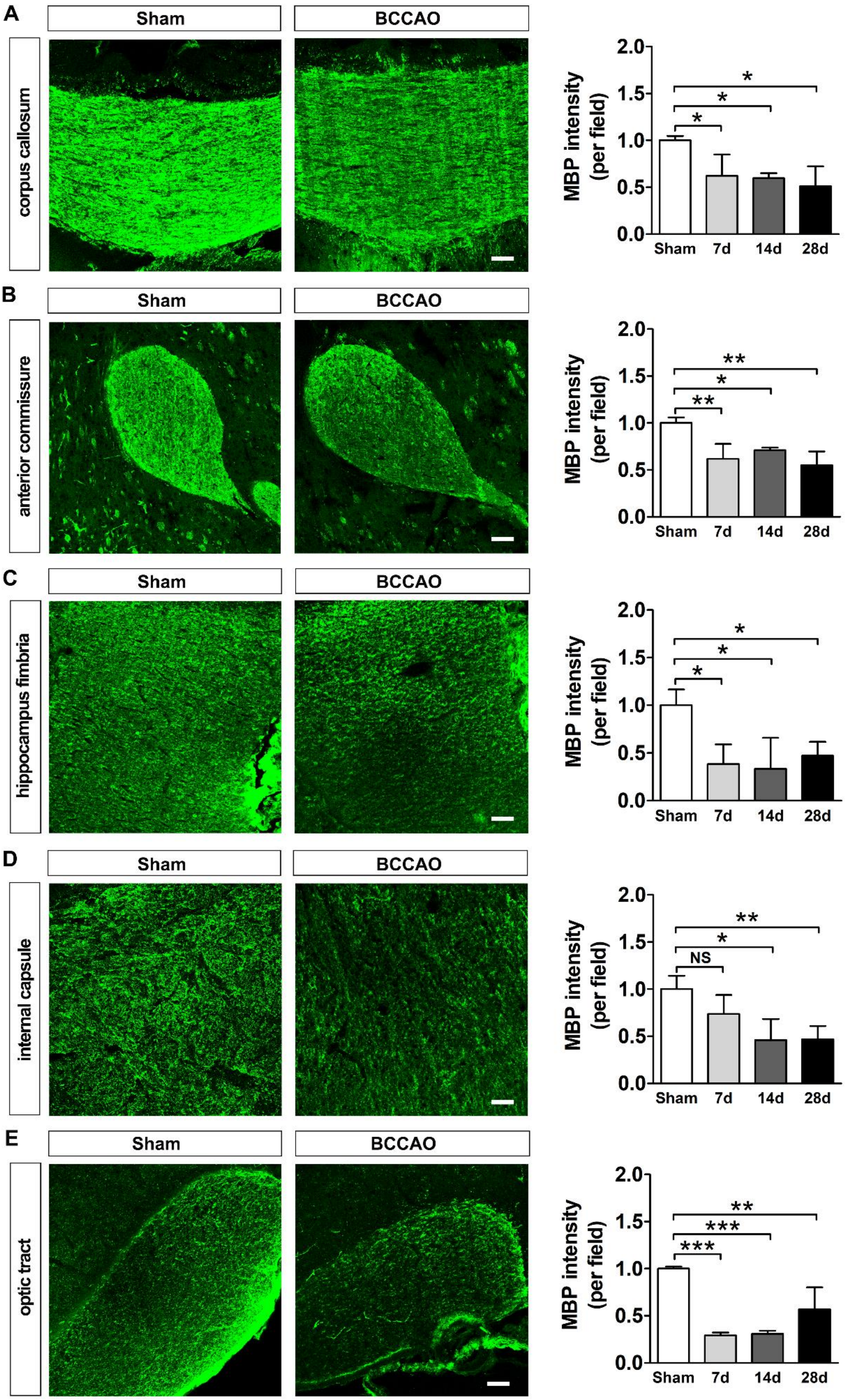
A
Sham
BCCAO
corpus callosum
MBP intensity (per field)
2.0
1.5
1.0
0.5
0.0
*
*
*
Sham
7d
14d
28d
B
Sham
BCCAO
anterior commissure
MBP intensity (per field)
**
*
**
Sham
7d
14d
28d
C
Sham
BCCAO
hippocampus fimbria
MBP intensity (per field)
*
*
*
Sham
7d
14d
28d
D
Sham
BCCAO
internal capsule
MBP intensity (per field)
**
*
NS
Sham
7d
14d
28d
E
Sham
BCCAO
optic tract
MBP intensity (per field)
**
***
***
Sham
7d
14d
28d

Figure S2 BCCAO-induced myelin degradation in different regions of white matter. A-E Representative images and quantification of MBP (green) fluorescence intensity in corpus callosum (**A**), anterior commissure (**B**), hippocampus fimbria (**C**), internal capsule (**D**), and optic tract (**E**) in the BCCAO rats and the control rats. Scale bar=50 μm. The data are shown as the mean ± SD. n = 3-4 animals in each group. ***, $p< 0.001$; **, $p< 0.01$; *, $p< 0.05$; NS, not significant. MBP: myelin basic protein.

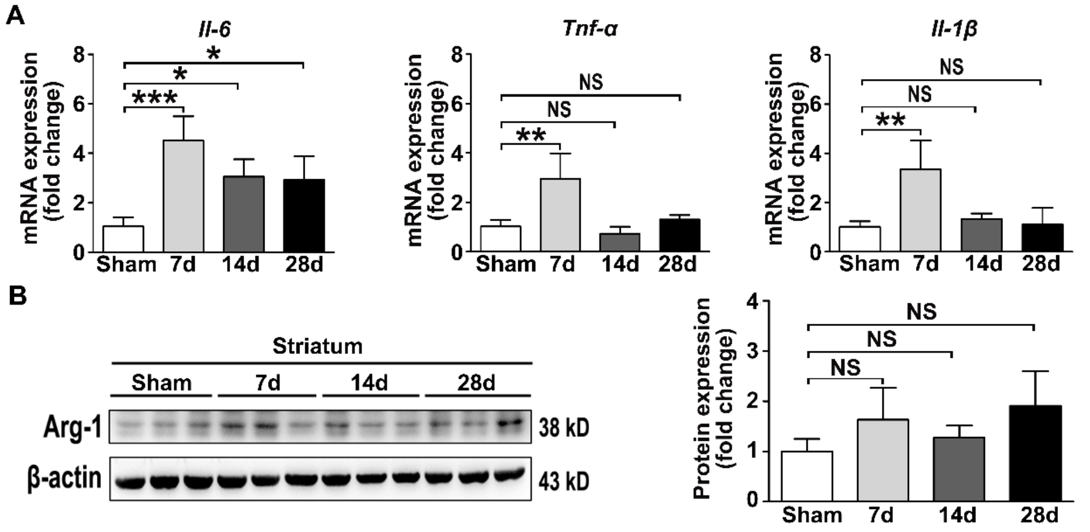

Figure S3 Up-regulation of the activated microglia-related pro-inflammatory cytokines with no change in the expression of the protective phenotype microglia marker in BCCAO rats. A Quantitative RT-PCR analysis of the expression of *Il-6*, *Tnf-α*, and *Il-1β* in the striatum of control and BCCAO rats at day 7, 14, and 28 after the surgery. The values are normalized to those of the sham group. n = 3-6 in each group. **B** Western blot and quantification for Arginase-1, β-actin in the striatum of BCCAO and control rats at day 7, 14, and 28 after surgery. n = 3 in each group. The data are shown as the mean ± SD. ***, $p < 0.001$; **, $p < 0.01$; *, $p < 0.05$; NS, not significant; the BCCAO group vs. the control group. Arg-1: Arginase-1.

A

Sequence of start and goal positions for assessing working memory

Day	Start	Goal
1	N	SE
2	E	NE
3	S	SW
4	W	SE

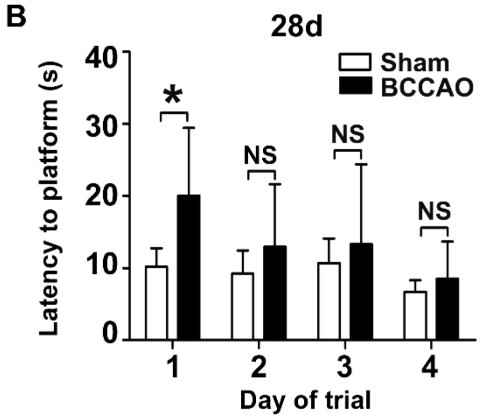

Figure S4 BCCAO-induced working memory impairments. A Sequence of start and goal positions for assessing working memory in the Morris water maze test in control and BCCAO rats at day 28 after surgery. **B** Four-day working memory performance measured as the latency to reach the platform in the Morris water maze test in control and BCCAO rats at day 28 after surgery. n = 12-15 animals in each group. The data are shown as the mean ± SD. *, $p< 0.05$; NS, not significant; the BCCAO group vs. the control group.

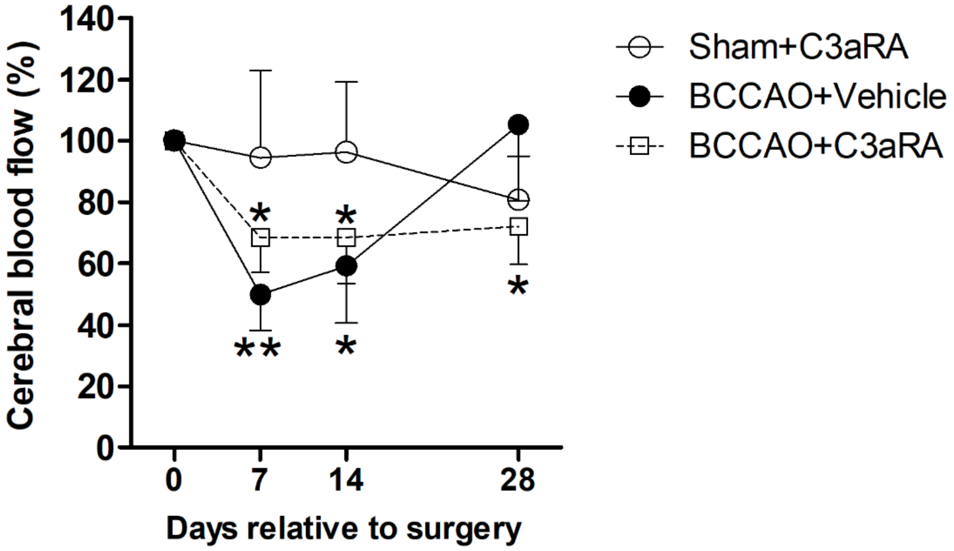

Figure S5 Dynamic CBF change under C3aR inhibition in BCCAO and control rats. Quantification of dynamic cerebral blood flow changes in the whole brain in BCCAO and control rats treated with C3aR antagonist or vehicle at day 7, 14, and 28 after surgery, relative to baseline levels. n = 4-6 animals. The data are shown as the mean ± SD. **, $p < 0.01$; *, $p < 0.05$; NS, not significant; different time point vs. baseline level. 3D ASL: 3D arterial spin-labeled imaging; C3aRA: C3aR antagonist.

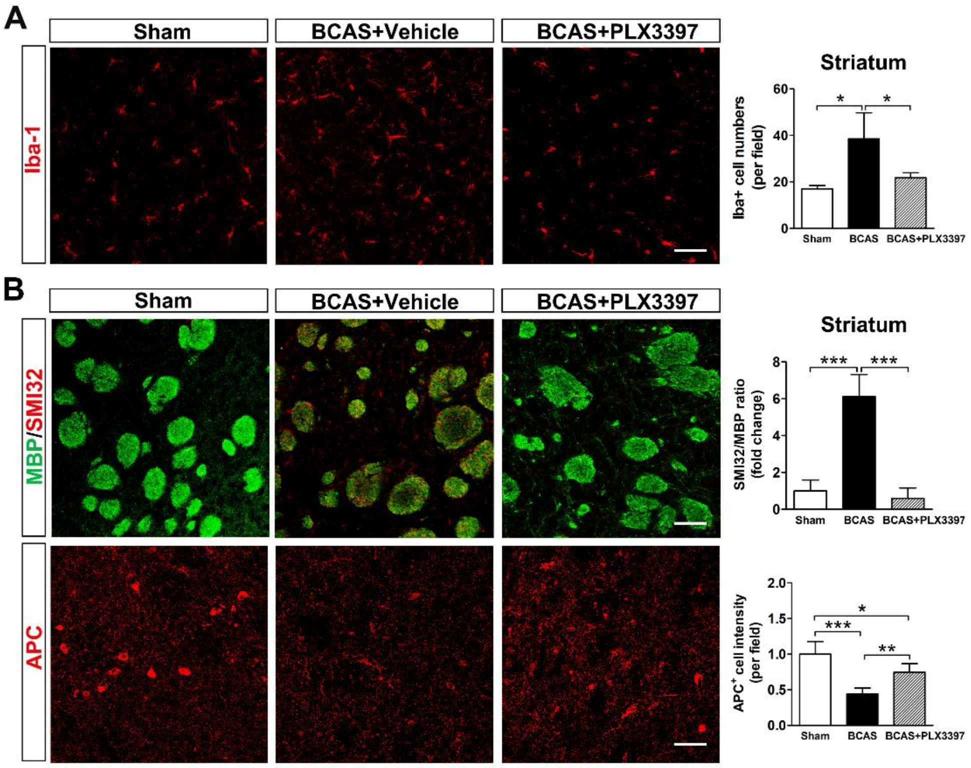

Figure S6 Microglia ablation by CSF1R inhibitor PLX3397 prevents white matter injury in BCAS mice. A Representative images and quantification for the number of Iba-1$^+$ microglia cells (red) in the striatum of the control, BCAS+vehicle, and BCAS+PLX3397 mice at day 28 after surgery. Scale bar=50 μm. **B** Representative images and quantification of damaged axon (SMI32$^+$, red) relative to myelin (MBP$^+$, green) and mature oligodendrocyte (APC$^+$ cells, red) in the striatum of the control, BCAS+vehicle, and BCAS+PLX3397 mice at day 28 after surgery. The data are shown as the mean ± SD. Scale bar=50 μm. n = 3-4 animals in each group. ***, $p< 0.001$; **, $p< 0.01$; *, $p < 0.05$.

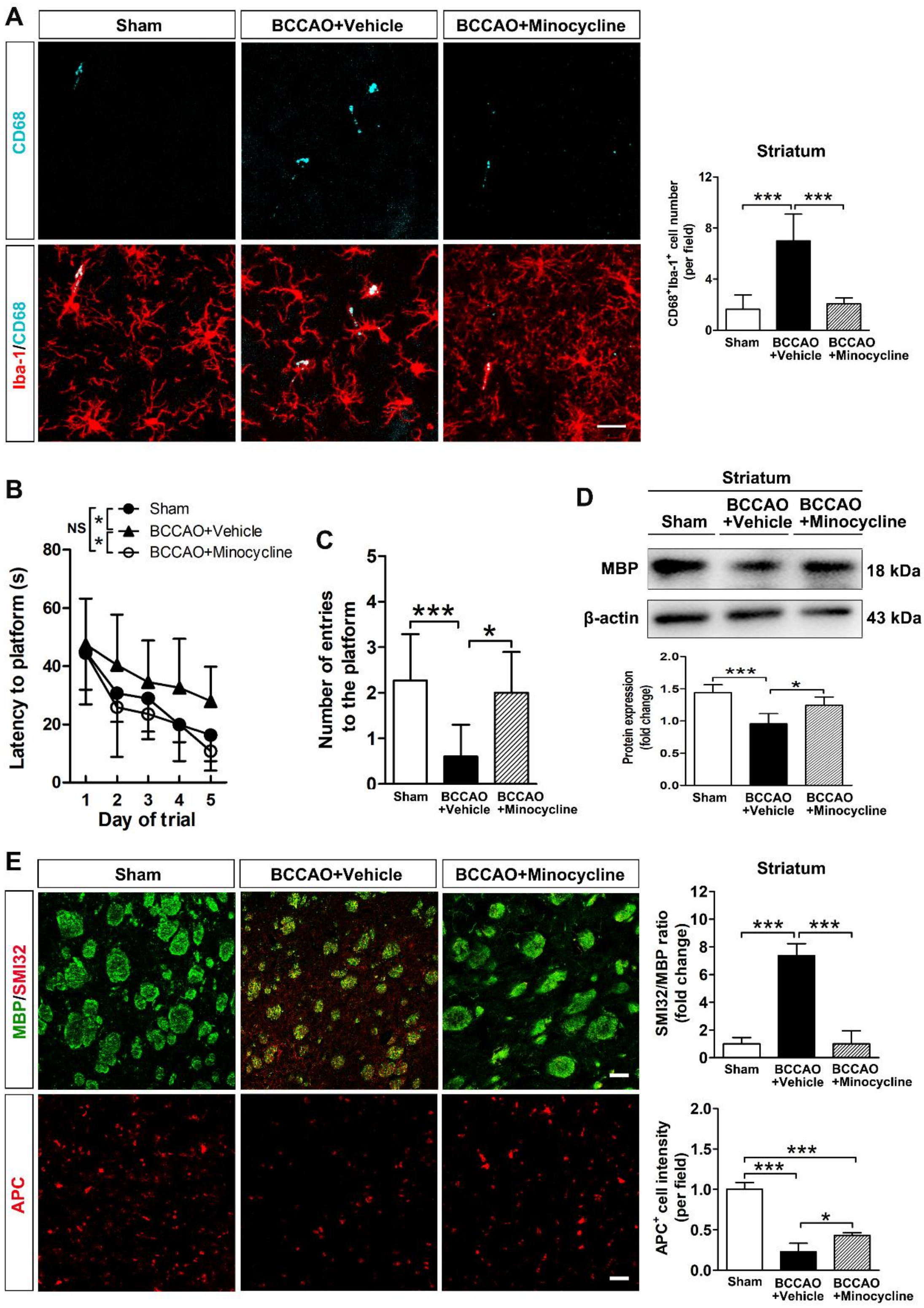

Figure S7 Microglia inactivation by minocycline prevents behavioral deficits and white matter injury in BCCAO rats. **A** Representative images and quantification for the number of activated microglia cells ($CD68^{+}$ and $Iba\text{-}1^{+}$ double positive cells,

indigo) in the striatum of the control, BCCAO+vehicle, and BCCAO+minocycline rats at day 28 after surgery. Scale bar=25 μm. **B** Five-day spatial learning performance measured as the latency to reach the platform in the Morris water maze test in the control, BCCAO+vehicle, and BCCAO+minocycline rats at day 28 after surgery. n = 6-15 animals for each group. **C** Spatial memory performance measured as the number of entries into the platform quadrant in the Morris water maze test in the control, BCCAO+vehicle, and BCCAO+minocycline rats at day 28 after surgery, n = 6-15 animals in each group. **D** Western blots and quantification for myelin basic protein (MBP) and β-actin in the striatum of the control, BCCAO+vehicle, and BCCAO+minocycline rats at day 28 after surgery. **E** Representative images and quantification of damaged axon ($SMI32^+$, red) relative to myelin (MBP^+, green) and mature oligodendrocyte (APC^+ cells, red) in the striatum of the control, BCCAO+vehicle, and BCCAO+minocycline groups. Scale bar=50 μm. The data are shown as the mean ± SD. n= 3-6 animals in each group unless otherwise noted. ***, $p< 0.001$; *, $p< 0.05$; NS, not significant.

Video legend

Suppl. Movie 1 CLARITY imaging of microglia distribution relative to myelin fibers in the striatum of control (left) and BCCAO (right) rat. Imaging of 500-μm-thick clarified rat brain slices co-stained with Iba-1 (red) and myelin basic protein (MBP, green) in control (**left**) and BCCAO rat (**right**). An even distribution of microglia relative to myelin fibers is shown in the striatum of the control rat. BCCAO rat shows more microglia in contact with myelin fibers in the striatum compared with the control. The volumes of three-dimensional rendering are 309 μm×309 μm×200 μm, with a voxel size of 1.01 μm×1.01 μm×1.00 μm.
